# Selenized Tripterine Phytosomes Alleviate Ferroptosis-Mediated Acute Kidney Injury by Suppressing GPX4 Degradation via the DUSP1/Autophagy Pathway

**DOI:** 10.34133/bmr.0236

**Published:** 2025-08-12

**Authors:** Liang Yan, Qi Feng, Yong Sun, Bo-ning Zeng, Chuan-chuan Sun, Qing-bing Zha, Xing-wang Zhang, Shi-ping Zhu

**Affiliations:** ^1^ Guangdong Provincial Key Laboratory of Spine and Spinal Cord Reconstruction, The Fifth Affiliated Hospital (Heyuan Shenhe People’s Hospital), Jinan University, Heyuan 517000, China.; ^2^ Department of Chinese Traditional Medicine, The First Affiliated Hospital of Jinan University, Guangzhou 510630, China.; ^3^ Department of Clinical Laboratory, The Fifth Affiliated Hospital (Heyuan Shenhe People’s Hospital), Jinan University, Heyuan 517000, China.; ^4^ Department of Medical Biochemistry and Molecular Biology, School of Medicine, Jinan University, Guangzhou 510632, China.; ^5^ School of Medicine, Jinan University, Guangzhou 510630, China.; ^6^ Department of General Practice, The 3rd Affiliated Hospital of Shenzhen University (Shenzhen Luohu People’s Hospital), Shenzhen 518001, China.; ^7^ Department of Nephrology, The First Affiliated Hospital of Jinan University, Guangzhou 510630, China.; ^8^ Department of Pharmaceutics, School of Pharmacy, Jinan University, Guangzhou 511443, China.

## Abstract

Ferroptosis, a form of lipid peroxidation-mediated cell death, plays a critical role in acute kidney injury (AKI) progression. Tripterine is an active component isolated from traditional medicinal herbs and exhibits diverse biological and pharmacological activities. However, poor bioavailability and cytotoxicity of tripterine has limited its further clinical application, and the underlying action mechanism of tripterine against AKI remains largely unknown. This study aimed to overcome these shortcomings by formulating tripterine into selenized phytosomes and to investigate the therapeutic effects of selenized tripterine phytosomes (Se@Tri-PTs) on ferroptosis-associated AKI. The data showed that Se@Tri-PTs improved the antioxidant capacity of tripterine while reducing its cytotoxicity. Upon erastin or RSL3 stimulation, Se@Tri-PTs maintained intracellular glutathione levels, decreased lipid ROS generation, and suppressed ferroptosis. Mechanistically, Se@Tri-PTs blocked autophagy-mediated degradation of glutathione peroxidase 4 (GPX4), thereby suppressing ferroptosis. Furthermore, Se@Tri-PTs maintained dual-specificity protein phosphatase 1 (DUSP1) protein levels in erastin-stimulated cells, and *DUSP1* knockdown reversed Se@Tri-PTs-mediated inhibition of autophagy and ferroptosis. In line with in vitro results, Se@Tri-PTs administration obviously attenuated folic acid-induced AKI and autophagy-dependent ferroptosis in mice. Collectively, these results indicated that Se@Tri-PTs ameliorated ferroptosis and AKI by preserving DUSP1 levels to block autophagy-mediated degradation of GPX4, highlighting their potential in treating ferroptosis-related diseases.

## Introduction

Acute kidney injury (AKI) is characterized by the sudden loss of renal function accompanied by an irreversible loss of kidney cells and nephrons, which can be induced by a variety of factors, including ischemia, hypovolemia, nephrotoxic chemicals, inflammation, and vasculitis [1]. Unfortunately, there are still no effective therapies and/or targeted treatment drugs for AKI. In addition, untreated AKI may continue to cause further renal damage, leading to poor prognosis [2]. Therefore, the exploration of new therapeutic targets or drugs for AKI has become an urgent demand.

The pathophysiology of AKI involves a variety forms of cell death, and ferroptosis has been shown to play a critical role in AKI progression [3]. Ferroptosis is a newly characterized form of regulated cell death mediated by iron-dependent accumulation of lipid reactive oxygen species (ROS). It is regulated by a variety of molecular mechanisms, including cystine/glutamate transporter (SLC7A11/xCT), glutathione peroxidase 4 (GPX4), lipid metabolism, and iron metabolism pathways [4]. Mechanistically, autophagy signaling plays a significant role in the induction of ferroptosis, in which some key proteins are degraded by autophagosome during ferroptosis [5]. For example, GPX4 degradation is induced under copper stress by the autophagy pathway, and subsequently, ferroptosis is triggered [6]. Consistently, Fin56-induced GPX4 degradation by autophagy can be blocked by autophagy inhibitor in mouse embryo fibroblasts and bladder cancer cells [7]. Furthermore, inactivation of the antiferroptosis GPX4 causes acute renal failure in mice, implicating ferroptosis in AKI [8]. Supporting this, legumain promotes tubular ferroptosis by facilitating chaperone-mediated autophagy (CMA) that leads to degradation of GPX4 in AKI [9]. These findings indicate that autophagy-mediated GPX4 degradation contributes to the biological process of ferroptosis, and it may be a cause of ferroptosis-induced AKI.

*Tripterygium wilfordii* is a traditional medicinal herb that has long been used for the treatment of nephrotic syndrome and glomerulonephritis, and tripterine (Tri) is a major active ingredient of this herbal medicine [10]. Tripterine has been shown to possess various pharmacological effects, including anti-inflammation, antiobesity, antitumor, and antioxidative activities [11]. In view of its potent antioxidative effect, tripterine has been demonstrated to significantly alleviate oxidative stress and vascular calcification in chronic kidney disease by up-regulating heme oxygenase-1 (HO-1) [12]. In addition, tripterine exhibited excellent protective effects against oxidative insults via activating the Nrf2 signaling pathway and up-regulating the expression of glutamate-cysteine ligase modifier subunit [13]. Recently, tripterine has been shown to alleviate cisplatin-induced AKI by inhibiting ferroptosis [14]. These previous studies suggested that tripterine may have potential therapeutic effects on ferroptosis-mediated AKI. However, the practical application of tripterine is highly challenged by its poor water solubility [15] and overt cytotoxicity [16]. In an attempt to reduce these shortcomings of tripterine, we had prepared selenized tripterine phytosomes (Se@Tri-PTs) and characterized their properties. We found that Se@Tri-PTs had reduced cytotoxicity, enhanced apparent solubility, and facilitated intracellular delivery, while increasing anti-inflammatory efficacy when compared with the free drug [17–19]. Although the therapeutic effect of tripterine on ferroptosis-associated disease has been reported recently [14], the effect of Se@Tri-PTs on AKI and the underlying action mechanism remain uninvestigated.

**Fig. 1.**
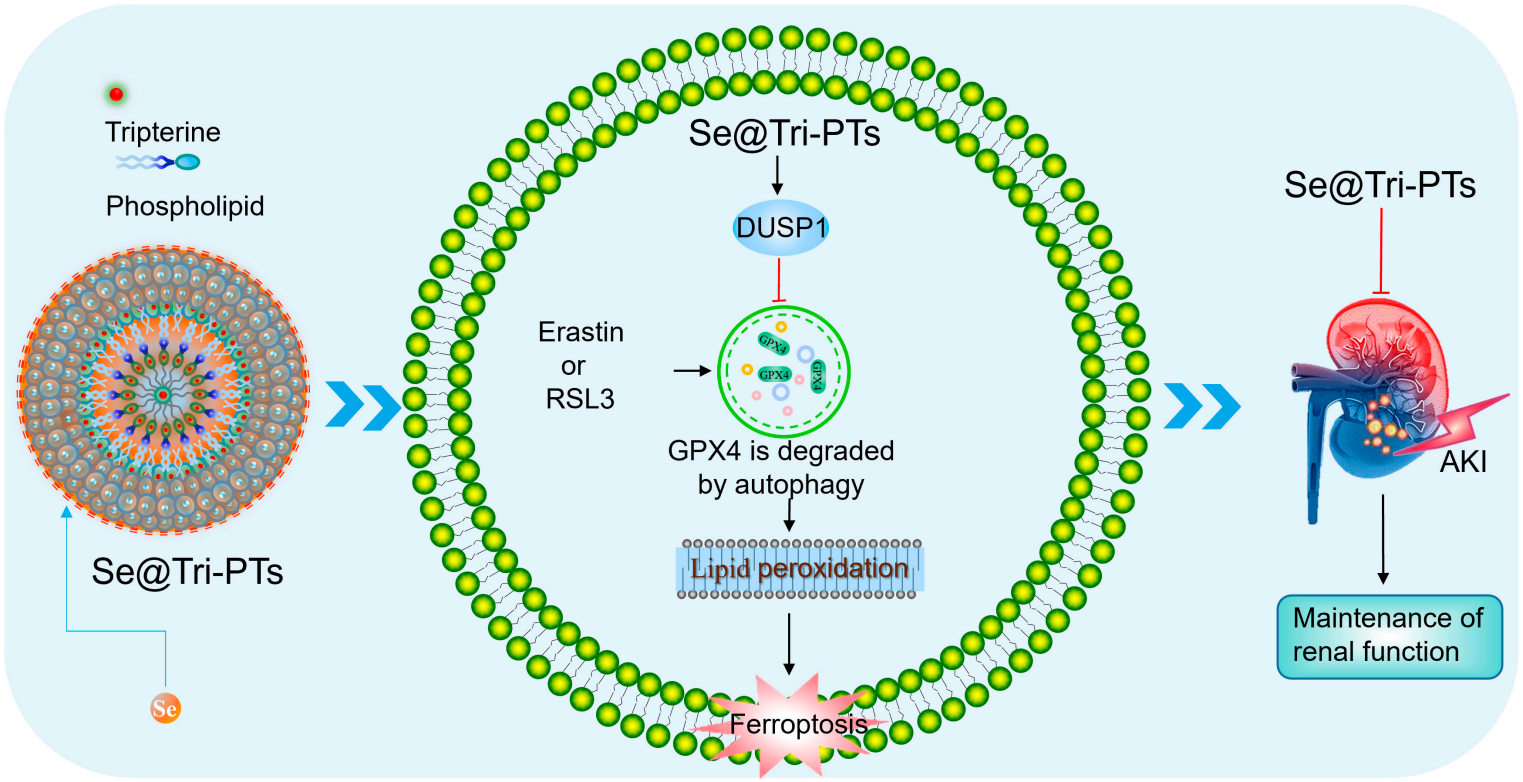
Schematic diagram of the protective effect of Se@Tri-PTs on ferroptosis and FA-induced AKI. Se@Tri-PTs might inhibit autophagy to block degradation of GPX4 via maintaining DUSP1 levels, leading to amelioration of ferroptosis and folic acid (FA)-induced AKI.

In the present study, we demonstrated that Se@Tri-PTs were able to decrease the cytotoxicity of tripterine on HK-2 cells while enhancing its antioxidant activity. Se@Tri-PTs inhibited erastin- and RSL3-induced ferroptosis, and the inhibitory effect of Se@Tri-PTs on ferroptosis was superior to that of Se@PTs (blank carrier) and free tripterine. Mechanistically, Se@Tri-PTs maintained dual-specificity protein phosphatase 1 (DUSP1) levels to block autophagy-mediated degradation of GPX4, thereby reducing lipid ROS production and ferroptosis. Moreover, Se@Tri-PTs was able to alleviate folic acid (FA)-induced AKI and ferroptosis in vivo. Collectively, our data revealed that Se@Tri-PTs-induced alleviation of AKI was associated with inhibition of ferroptosis via regulating the DUSP1/autophagy/GPX4 axis (Fig. 1).

## Materials and Methods

### Reagents and antibodies

Tripterine was purchased from DASF Biological Technology Co. (Nanjing, China). The ferroptosis activator erastin and RSL3 were bought from TopScience (Shanghai, China). Soybean lecithin (Lipoid S100, phosphatidylcholine ≥ 94%) was provided by Lipoid GmbH (Ludwigshafen, Germany). Sodium oleate, sodium selenite (Na_2_SeO_3_), and reduced glutathione (GSH) were obtained from Aladdin (Shanghai, China). 3,3′-Dioctadecyloxacarbonyl cyanine perchlorate (DiO), FA, and Hoechst 33342 were purchased from Sigma-Aldrich (St. Louis, MO, USA). Dulbecco’s modified eagle medium/nutrient mixture F-12 (DMEM/F-12), fetal bovine serum (FBS), Opti-MEM, and BODIPY 581/591 C11 were products of Thermo Fisher (Carlsbad, CA, USA). The antibodies against β-actin, tubulin, P62, LC3B, adenosine 5′-monophosphate (AMP)-activated protein kinase (AMPK), p-AMPK, mammalian target of rapamycin (mTOR), p-mTOR, horseradish peroxidase (HRP)-linked horse anti-mouse IgG, and HRP-linked goat anti-rabbit IgG were purchased from Cell Signaling Technology (Danvers, MA, USA). Anti-DUSP1, anti-GPX4, and anti-SLC7A11 (xCT) antibodies and rapamycin were procured from Selleck Chemicals (Houston, TX, USA). GSH, oxidized glutathione (GSSG), and malondialdehyde (MDA) assay kits were bought from Beyotime (Shanghai, China). The anti-4-HNE antibody was a product of R&D Systems (Emeryville, CA, USA). Creatinine and blood urea nitrogen (BUN) assay kits were from Nanjing Jiancheng Bioengineering Institute (Nanjing, China).

### Cell line

HK-2 cells and HEK293 cells were obtained from the Cell Bank of the Chinese Academy of Sciences (Shanghai, China). HK-2 cells were maintained in complete DMEM/F12 medium and HEK293 cells were cultured in complete DMEM medium (containing 10% FBS, 100 U/ml of penicillin, and 100 μg/ml of streptomycin) at 37 °C in a humidified incubator with 5 % CO_2_ and sub-cultured every 2 to 3 days.

### Preparation and characterization of Se@Tri-PTs

Se@Tri-PTs were prepared as previously reported [17]. In brief, tripterine and soybean lecithin were weighed at a stoichiometric ratio of 1:1, and then an appropriate quantity of tetrahydrofuran was used to dissolve them. Subsequently, the organic solvent was evaporated out using a rotary evaporator under a reduced pressure. After removal of the solvent, the mixture containing tripterine and phospholipid molecules was allowed to react for 1 h at 55 °C to enable conjugation under the protection of N_2_. Then, the resultant complexes were hydrated against Na_2_SeO_3_ solution at 55 °C. When the phospholipid complexes were dislodged from the flask wall, the crude dispersions were subjected to sonication for 2 min at 100 W to obtain Tri-PTs. To prepare Se@Tri-PTs, a 4-fold molar amount of reduced GSH (equivalent to Na_2_SeO_3_) was introduced into Tri-PTs. The redox was proceeded for 2.5 h at 37 °C under agitation. Se@Tri-PTs were generated when Se^4+^ was reduced into elemental Se that deposited inside and outside Tri-PTs. The residual reactants were removed by dialysis against deionized water twice.

The particle size and zeta potential of Se@Tri-PTs and other counterparts were measured using a Malvern analyzer (Nano ZS, Worcestershire, UK) at 25 °C. The micromorphology of Se@Tri-PTs was observed via transmission electron microscopy (TEM) (Tecnai 10, Philips, Eindhoven, Netherlands). The release of tripterine at different time points from Se@Tri-PTs or Tri-PTs was measured by using ultraviolet–visible (UV–Vis) spectroscopy. Detailed experimental procedures were described as previously reported [17].

### Cellular uptake

HK-2 cells were seeded in 6-well plates and maintained at 37 °C in a humidified incubator overnight. Then, the cells were indicated with DiO-labeled Tri-PTs or Se@Tri-PTs for 2 h at 37 °C. Subsequently, the cells were rinsed by sterile phosphate-buffered saline (PBS) followed by nuclei staining with Hoechst 33258 for 10 min at 37 °C. Live imaging was performed immediately by using a Zeiss Axio Observer D1 microscope and fluorescence images were captured with a Zeiss Axio cam MRR3 cooled CCD camera (Carl Zeiss, Oberkochen, Germany).

### Cell viability assay

Cell viability was detected by the Cell Counting Kit-8 (CCK-8) assay. The cells in logarithmic phase were seeded in 96-well plates at a density of 0.8 × 10^4^ cells per well and were maintained at 37 °C in a humidified incubator overnight. Cells were treated with Se@ PTs, Tri, or Se@Tri-PTs for 48 h, and then 10 μl of CCK-8 reagent was added to each well. The plates were further incubated for 2 h at 37 °C. The optical density (OD) values were measured at 450 nm using a Spectra Max iD5 microplate reader (Molecular Devices, San Jose, USA).

### Cell death assay

The cells were seeded in 6-well plates and then treated with Se@PTs, Tri, or Se@Tri-PTs with/without erastin or RSL3 for the indicated time periods. Then, they were observed immediately by live imaging and the images were captured with a Leica microscope DMi8-M camera (Leica, Germany).

### Determination of lipid ROS

Cells were seeded on 6-well plates and incubated overnight. The next day, cells were treated with Se@PTs, Tri, or Se@Tri-PTs with/without erastin or RSL3 for the indicated time periods, and then the culture medium was removed and replaced with fresh culture medium containing 5 μM of C11-BODIPY 581/591 (Invitrogen) for 1 h. Subsequently, the cells were harvested by trypsinization and resuspended in 400 μl of PBS. Finally, lipid peroxidation was assessed using a flow cytometer (NovoCyte2060R, Thermo Scientific, Waltham, USA) and analyzed using the FlowJo software.

### Real-time PCR analysis

Cells were treated with Se@Tri-PTs with/without erastin or RSL3 for the indicated time periods, and then they were extracted using Trizol (Invitrogen), and reverse transcribed into cDNA using a cDNA synthesis kit (Takara). The cDNA levels were measured by SYBR green real-time in the Light-cycler. The housekeeping gene β-actin was used for normalization in each individual sample and the 2^−ΔΔCt^ method was used to quantify relative expression changes. The sequences of specific primers used for quantitative reverse transcription polymerase chain reaction assay for GPX4 were 5′-GAGGCAAGACCGAAGTAAACTAC-3′ (forward) and 5′-CCGAACTGGTTACACGGGAA-3′ (reverse).

### Immunofluorescence analysis

Immunofluorescence analysis was performed as previously described [20]. In brief, HK-2 cells were seeded in glass-bottomed dishes and cultured at 37 °C overnight. The next day, the cells were treated with Se@Tri-PTs with/without erastin or RSL3 for the indicated time periods and then fixed with 4% paraformaldehyde for 15 min. After permeabilization and blocking, cells were incubated with anti-LC3II antibody overnight at 4 °C, followed by staining with CF568-conjugated goat-anti-rabbit IgG. Subsequently, cells were stained with Hoechst 33342 solution. Finally, they were observed under the Leica TCS SP8 confocal microscope (Wetzlar, Germany).

### RNA-seq profiling

RNA-seq profiling was performed by Novogene (Beijing, China). In brief, cells were treated with Se@Tri-PTs with/without erastin for 24 h, and then cellular RNA was isolated using the Trizol reagent. A total amount of 3 μg of RNA per sample was used as input material for the RNA sample preparation. Sequencing libraries were generated using the NEB Next Ultra RNA Library Prep Kit for Illumina (NEB, Ipswich, MA, USA) following the manufacturer’s protocol. PCR products were purified (AMPure XP system) and library quality was assessed on the Agilent Bioanalyzer 2100 system. The differentially expressed genes were analyzed by the edge R package. Transcript expression with |Log2 Fc| > 1.0 and adjusted *p* value < 0.05 was regarded as statistically significant. All genes detected are shown in the Supplementary Materials.

### siRNA transfection

For transfection of siRNAs, cells were seeded in 6-cm dishes and cultured overnight. Until the cell confluency reached 60% to 80%, the cells were transfected with DUSP1 siRNA or control siRNA (Gene Pharma, China) using Lipo6000 transfection reagent (Beyotime) according to a protocol provided by the manufacturer.

### CRISPR/Cas9-mediated GPX4 knockout

The GPX4 depleted cells were generated with CRISPR/Cas9-mediated knockout system, using the Lenti-CRISPR V2 vector. The sgRNA sequences targeting human GPX4 were #1 5′-GCGCCCACCGGTACTTGTCC-3′ and #2 5′-GCGCACAGCGCCAGTCGTCC-3′. Lentivirus was produced by cotransfection of the lentiviral vector with psPAX and pMD2.G into 293T cells using polyethylenimine transfection reagent. HK-2 cells were seeded in 6-well plates followed by infecting with Lenti-CRISPRV2 viral supernatant containing 5 μg/ml of polybrene. Cells were selected with 5 μg/ml of puromycin 48 h after infection. Western blotting was used to detect the expression of the target protein and the infection efficiency.

### Western blot analysis

Western blot analysis was performed as previously described [21]. The proteins were dissolved with 1× sample loading buffer and separated by sodium dodecyl sulfate polyacrylamide gel electrophoresis (SDS-PAGE), followed by electro-transferring to polyvinylidene fluoride membranes (Millipore, Merck, MA, USA). The membranes were blocked in 5% skimmed milk blocking buffer and then incubated with the primary antibody overnight at 4 °C. Subsequently, they were incubated with the secondary antibody for 1 h at room temperature. The blot images were captured by the Tanon 5200 automatic chemiluminescence imaging system (Tanon, China).

### Analysis of GSH and MDA levels and renal function

GSH and GSSG assay kits and lipid peroxidation (reflected by MDA) assay kits were used to measure the levels of GSH and MDA in cell lysis or tissues according to the manufacturer’s instructions. The OD values were measured by using a Spectra Max iD5 microplate reader.

For renal function, creatinine assay kit and BUN assay kit were used to detect the levels of serum creatinine and BUN following the manufacturer’s protocol.

### FA-induced AKI model

Female C57BL/6J mice (7 to 8 weeks of age) were bought from GemPharmatech (Guangdong, China) and used for all studies. All animals were acclimatized for 1 week before experiments under 12-h dark/12-h light cycle conditions. Animal experiments were performed according to the guidelines for the care and use of animals approved by the Committee on the Ethics of Animal Experiments of Jinan University.

All mice were randomly divided into 3 groups and were intragastrically administered once a day for 3 consecutive days with Se@Tri-PTs (10 mg/kg body weight) or vehicle (PBS). FA-induced AKI was established by a single intraperitoneal injection of 200 mg/kg FA in 0.3 mol/l sodium bicarbonate as reported previously [22]. Mice were intragastrically administered once again with Se@Tri-PTs or vehicle 1 h after FA treatment. All mice were euthanized at 24 h after drug treatment. Blood samples and kidney tissues were collected at the time of euthanasia. For every mouse, one kidney was fixed in 4% phosphate-buffered formaldehyde, and the other one was snap frozen and saved at −80 °C.

### Histology analysis of kidney tissue

Kidney sections were stained with Masson staining or hematoxylin and eosin (H&E) to observe the tubular injury, including cell injury (loss of brush border or vacuolization), cell desquamation, and tubular dilation and signs of regeneration. The images were captured by the Leica microscope. Tubular injury was semiquantitatively scored by the percentage of damaged tubules in H&E staining: 0: no damage; 1: <25%; 2: 25% to 50%; 3: 50% to 75%; and 4: >75%. Renal fibrosis was assessed as the percentage of collagen deposition area over the total renal area by Masson staining.

### Immunohistochemical analysis

VECTASTAIN Universal Quick Kit, Avidin/Biotin Blocking Kit, and DAB peroxidase substrate assay kit were used for immunohistochemical analysis according to the manufacturer’s instructions. The images were captured by the Leica microscope.

### Statistical analysis

Each experiment was performed at least 3 times independently. Data were presented as mean ± standard deviation (SD). Statistical analysis was performed using GraphPad Prism 5.0. One-way analysis of variance followed by Tukey’s post hoc test and unpaired Student’s *t* test were used to analyze the statistical significance among multiple groups or between 2 groups. *P* < 0.05 was considered statistically significant (ns, *P* > 0.05; *P < 0.05; ** *P* < 0.01; *** *P* < 0.001).

## Results

### Characterization and cellular uptake of Se@Tri-PTs

**Fig. 2.**
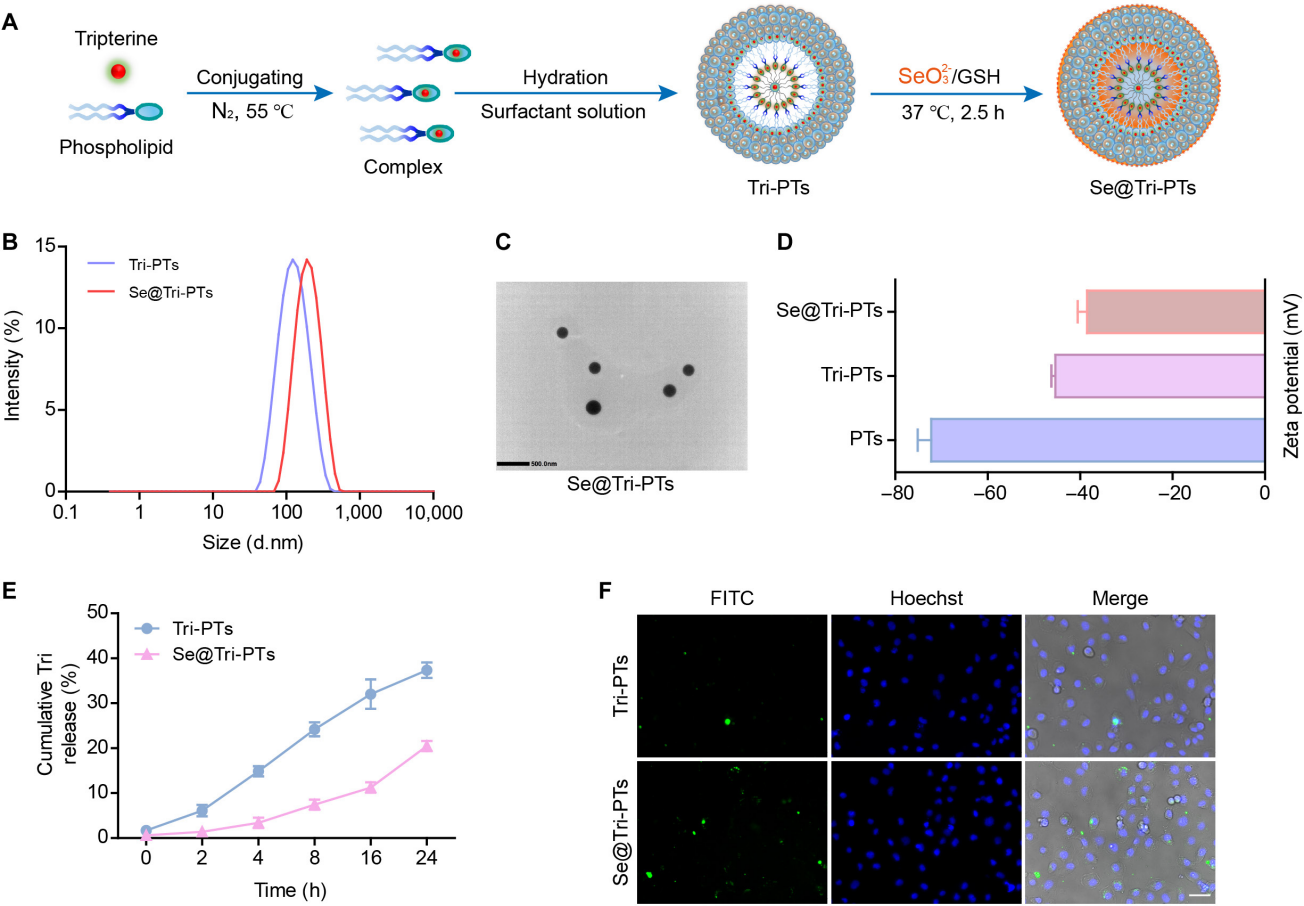
Characterization and cellular uptake of Se@Tri-PTs. (A) Preparation and structural illustration of Se@Tri-PTs. (B) Particle size distribution determined by dynamic light scattering (DLS). (C) Micromorphology of Se@Tri-PTs observed by TEM (scale bar: 500 nm). (D) Zeta potentials of PTs, Tri-PTs, and Se@Tri-PTs by DLS. (E) Time-dependent Tri releasing from Tri-TPs and Se@Tri-PTs by UV–Vis. (F) Representative immunofluorescence images showing the uptake levels of Se@Tri-PTs or Tri-PTs in HK-2 cells after incubation for 2 h. Tri, tripterine; Tri-PTs, tripterine phytosomes; Se@Tri-PTs, selenized tripterine phytosomes.

Tripterine is highly water-insoluble, making it difficult to be applied in practice. Phytosomes (PTs) can not only solve the solubility challenge but also promote drug absorption by the intestine. In our study, tripterine was chemically conjugated onto phospholipid molecules followed by self-assembly into nanovesicles in an aqueous medium (Fig. 2A). To further functionalize the nanomedicine, selenization was carried out by in situ reduction of Se^4+^ with GSH onto the surface of PTs, and then Se@Tri-PTs were obtained (Fig. 2A). The particle size of Se@Tri-PTs was around 200 nm, which was slightly larger than nonselenized tripterine phytosomes (Tri-PTs) (Fig. 2B). Through TEM imaging, the particles of Se@Tri-PTs were also determined to be ~200 nm in size (Fig. 2C), with all of them exhibiting spherical morphology. Moreover, zeta potential measurements indicated zeta potentials of −72, −45, and −38.5 mV for PTs, Tri-PTs, and Se@Tri-PTs, respectively (Fig. 2D). Additionally, UV–Vis results showed that tripterine could be released gradually, while Se@Tri-PTs could slow the process compared to Tri-TPs (Fig. 2E), indicating that Selenium (Se) on Se@Tri-PTs might reduce the unnecessary release of drug. Finally, the uptake of Se@Tri-PTs in HK-2 cells was detected, and our data showed that Se@Tri-PTs were more readily taken up by the cells when compared to Tri-PTs (Fig. 2F).

### Se@Tri-PTs inhibit ferroptosis in renal cells

**Fig. 3.**
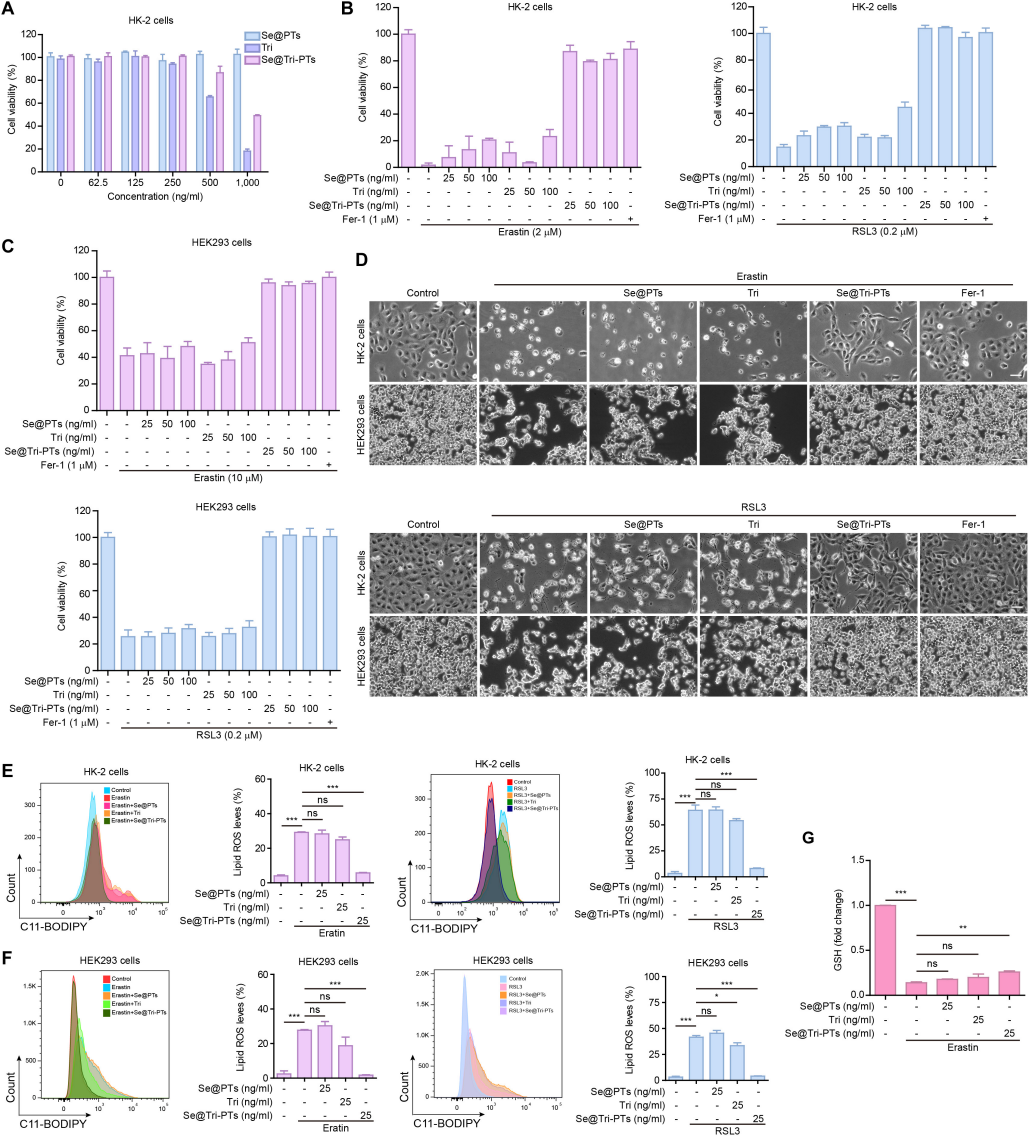
Se@Tri-PTs significantly inhibit erastin- or RSL3-induced ferroptosis. (A) Cell viability was detected by CCK-8 after treatment with Se@PTs, Tri, or Se@Tri-PTs for 48 h. (B) The HK-2 cells were coincubated with erastin for 24 h or RSL3 for 12 h in the presence of graded concentrations of Se@PTs, Tri, or Se@Tri-PTs, and then cell viability was detected by CCK-8. (C) The HEK293 cells were coincubated with erastin for 24 h or RSL3 for 24 h in the presence of graded concentrations of Se@PTs, Tri, or Se@Tri-PTs, and then cell viability was detected by CCK-8. (D) The representative live images showing the levels of cell death (scale bar: 50 μm). (E and F) Lipid ROS was detected by flow cytometry based on C11-BODIPY in HK-2 cells (E) or HEK293 cells (F), and the ratios of lipid ROS are shown on the right. (G) The HK-2 cells were treated with different medicaments for 24 h, and then GSH levels were measured. Data are expressed as mean ± SD (*n* = 3); *P < 0.05; **P < 0.01; ***P < 0.001; ns, not significant. Tri, tripterine; Se@PTs, selenized phytosomes; Se@Tri-PTs, selenized tripterine phytosomes.

The differences in pharmacological activity between Se@Tri-PTs, Se@PTs, and tripterine were next assessed. CCK-8 assay demonstrated that cell viability was decreased with the high doses of medicaments (500 and 1,000 ng/ml), indicating that tripterine itself has a certain cytotoxicity on HK-2 cells (Fig. 3A). Se@Tri-PTs had attenuated the cytotoxicity of tripterine on the cells (Fig. 3A). This reduced cytotoxicity might be due to the delayed release of tripterine from Se@Tri-PTs and might also be due to a function of Se in reducing the toxicity of drugs [23].

Due to the complexity of AKI pathogenesis, increasing evidence shows that ferroptosis is one of the factors that induce AKI. As commonly used in cellular models, the HK-2 cells or HEK293 cells were treated with erastin or RSL3 to induce ferroptosis. CCK-8 assay was used to detect cell viability, and the result showed that erastin or RSL3 treatment significantly decreased cell viability, which could be obviously reversed by ferroptosis inhibitor feroostatin-1 (Fer-1) (Fig. 3B and C). Notably, Se@Tri-PTs robustly suppressed erastin- or RSL3-induced decrease in cell viability, and its inhibitory effects were comparable to Fer-1 (Fig. 3B and C). However, upon erastin or RSL3 treatment, Se@PTs or free tripterine had only a weak inhibitory effect at high concentrations (Fig. 3B and C). Meanwhile, cell death was monitored via microscopy. As shown in Fig. 3D, cell death was rapidly induced by erastin or RSL3 treatment, which could be blocked by Fer-1. Consistent with the effect on cell viability, Se@Tri-PTs markedly inhibited erastin- or RSL3-induced cell death, though the cell proliferation appeared to be partially inhibited (Fig. 3D). Further supporting this, the proportion of erastin- or RSL3-induced lipid ROS (one of the classic features of ferroptosis) was ~30% or ~70% in HK-2 cells, respectively. However, levels of lipid ROS were ~10% in the group of Se@Tri-PTs cotreatment with erastin or RSL3 (Fig. 3E), which was also observed in HEK293 cells (Fig. 3F**)**. GSH is an important intracellular antioxidant, and Se@Tri-PTs could also recover the GSH level to a certain extent (Fig. 3G). Taken together, these results indicate that Se@Tri-PTs can markedly inhibit erastin- or RSL3-induced ferroptosis, while Se@PTs and tripterine only have a weak effect on ferroptosis.

### Se@Tri-PTs inhibit ferroptosis by blocking the degradation of GPX4 and autophagy

**Fig. 4.**
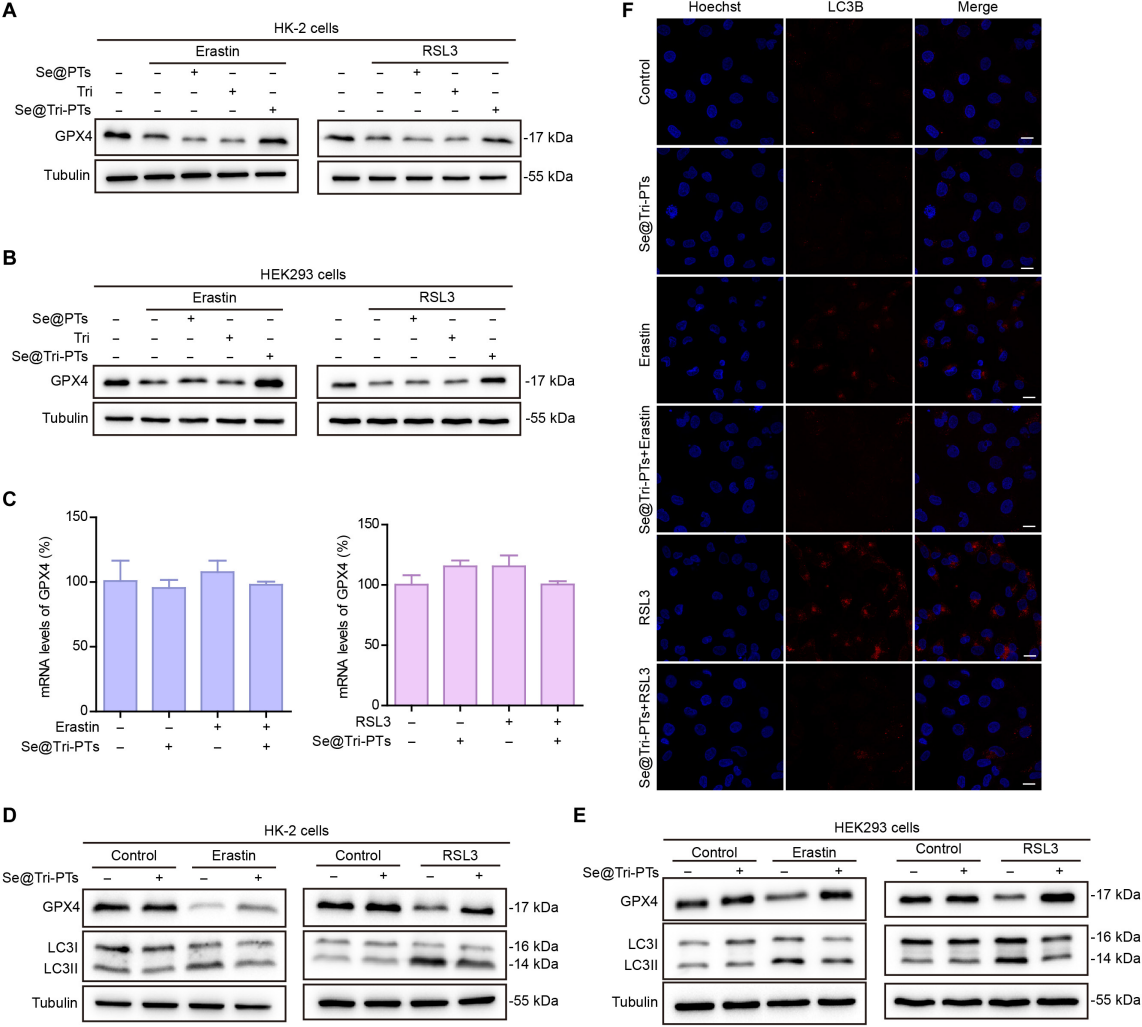
Se@Tri-PTs block erastin- or RSL3-induced degradation of GPX4 by inhibiting autophagy. (A and B) The cells were treated with erastin or RSL3 in the presence of graded concentrations of Se@PTs, Tri, or Se@Tri-PTs, and Western blotting was used to detect the expression of indicated proteins. (C) The mRNA levels of GPX4 were analyzed by RT-PCR. (D and E) The expression of indicated proteins was quantified by Western blotting. (F) The representative immunofluorescent images on the subcellular distribution of LC3II (red) with nuclei staining by Hoechst 33342 (blue) (scale bars: 10 μm). Tri, tripterine; Se@PTs, selenized phytosomes; Se@Tri-PTs, selenized tripterine phytosomes.

Erastin and RSL3 can induce ferroptosis by inhibiting SLC7A11 (xCT) and GPX4, respectively, in which inhibition of xCT by erastin decreases intracellular GSH to limit the capacity of GPX4 to prevent lipid peroxidation while RSL3 directly inhibits GPX4. Based on the above results that Se@Tri-PTs were able to inhibit ferroptosis in HK-2 and HEK293 cells, we next explored the molecular mechanism for Se@Tri-PTs to inhibit ferroptosis. Our results showed that both erastin and RSL3 decreased the expression of GPX4 (Fig. 4A and B), which could not be reversed by coincubation with Se@PTs or tripterine, whereas cotreatment with Se@Tri-PTs recovered the expression of GPX4 in erastin- or RSL3-treated cells (Fig. 4A and B). However, Se@Tri-PTs, Se@PTs, or tripterine could not reverse the erastin-induced decrease of xCT (Fig. S1). Intriguingly, we found that erastin, RSL3, or Se@Tri-PTs alone or their combination did not affect the mRNA expression of GPX4 (Fig. 4C), suggesting that the decrease of GPX4 levels might be due to protein degradation.

Recently, it has been found that the degradation of GPX4 can be induced by autophagy [6,7]. Therefore, we further explored the effect of Se@Tri-PTs on autophagy in HK-2 or HEK293 cells. As shown in Fig. 4D and E, erastin or RSL3 treatment alone could increase the expression levels of LC3II (indicative of elevated autophagy), but Se@Tri-PTs cotreatment could decrease the levels of LC3II back to levels of controls. Together, these results suggest that Se@Tri-PTs inhibit erastin- or RSL3-induced degradation of GPX4, which was associated with inhibition of autophagy.

### Se@Tri-PTs-mediated inhibition of ferroptosis is reversed by autophagy agonist

**Fig. 5.**
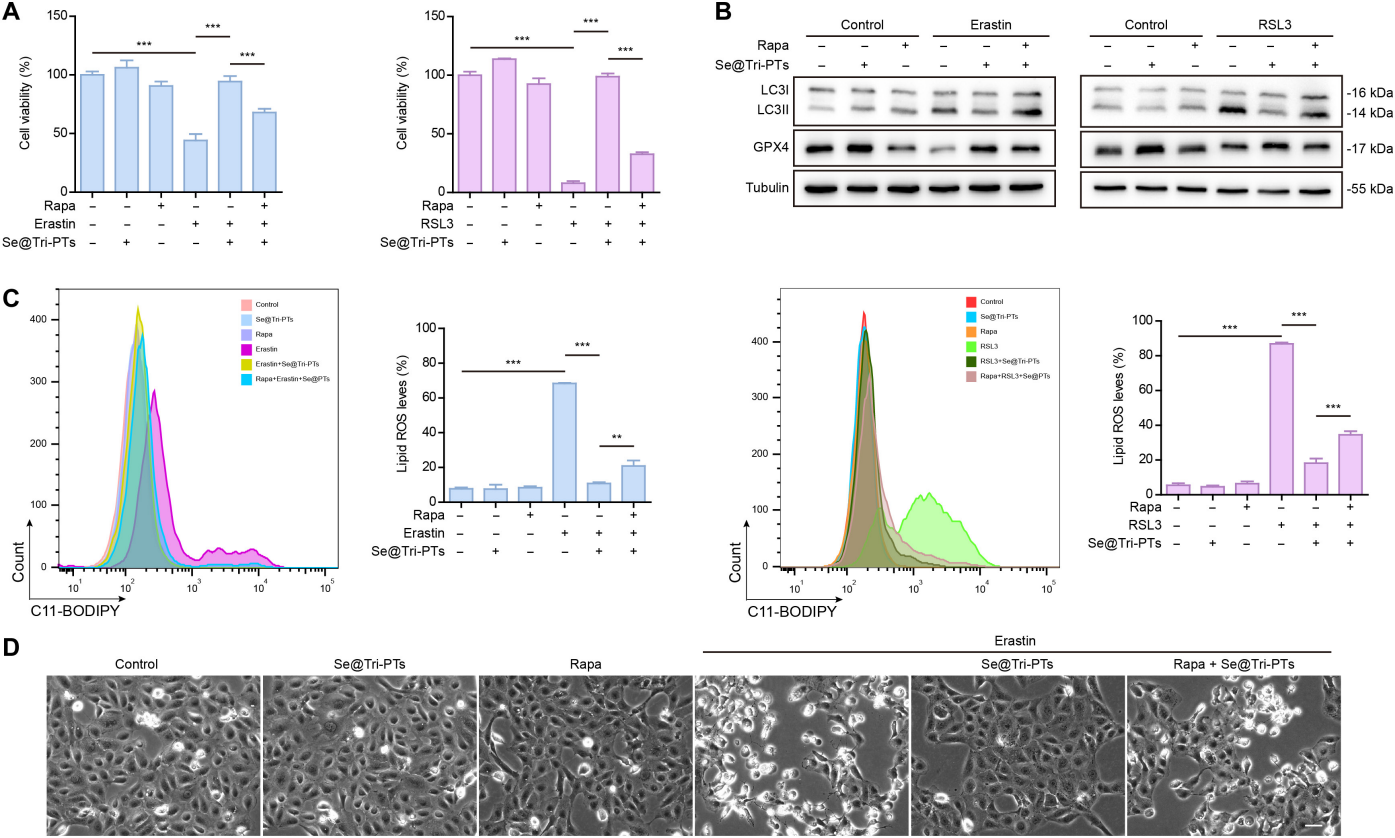
Activation of autophagy suppresses Se@Tri-PTs-mediated inhibition of ferroptosis. (A) The cells were incubated with Se@Tri-PTs and erastin for 18 h or RSL3 for 6 h, and then cotreated with the autophagy activator rapamycin (Rapa) for 6 h. CCK-8 assay was used to detect the cell viability. (B) Cells were treated as described in (A), and the expression of proteins was detected by Western blotting. (C) The lipid ROS was detected by flow cytometry using C11-BODIPY and expressed as the ratio of lipid ROS in histograms. (D) The representative live images showing the levels of cell death (scale bar: 50 μm). Data are expressed as mean ± SD (*n* = 3); **P < 0.01; ***P < 0.001.

To further confirm that Se@Tri-PTs maintain GPX4 levels by inhibiting autophagy in erastin- or RSL3-treated cells, the autophagy agonist rapamycin was used to induce autophagy. The results showed that Se@Tri-PTs-mediated inhibition of erastin- or RSL3-induced decrease of cell viability was significantly attenuated by rapamycin cotreatment (Fig. 5A). In line with this, rapamycin had an antagonistic effect on Se@Tri-PTs-induced increase of GPX4 and decrease of LC3II (Fig. 5B). Further, rapamycin reversed Se@Tri-PTs-mediated inhibition of erastin- or RSL3-induced production of lipid ROS (Fig. 5C). Besides, live imaging revealed that inhibition of cell death by Se@Tri-PTs was reversed by cotreatment with rapamycin (Fig. 5D). Together, these data further confirmed that the Se@Tri-PTs-mediated inhibition of erastin- or RSL3-induced GPX4 degradation and ferroptosis was accomplished by suppressing autophagy.

### GPX4 is the key switch for Se@Tri-PTs-mediated inhibition of ferroptosis

**Fig. 6.**
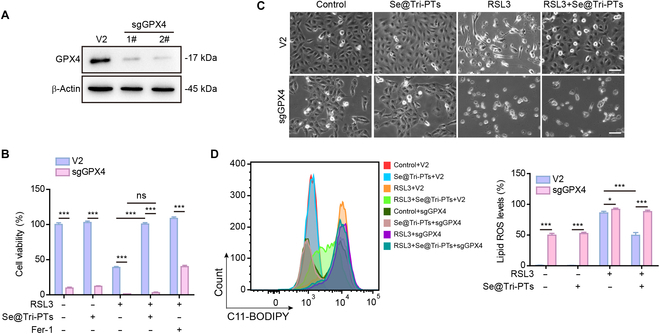
Se@Tri-PTs-mediated inhibition of ferroptosis depends on GPX4. (A) Knockout of GPX4 was performed by CRISPR/Cas9 in HK-2 cells, and the protein level of GPX4 was detected by Western blotting. (B) The cells were treated with or without Se@Tri-PTs, and coincubated with RSL3 for 12 h. CCK-8 assay was used to detect cell viability. The group of ferrostatin-1 (Fer-1) and RSL3 cotreatment was used as a control. (C) The representative live images showing the levels of cell death (scale bar: 50 μm). (D) The lipid ROS was detected by flow cytometry using C11-BODIPY. Data are expressed as mean ± SD (*n* = 3); *P < 0.05; ***P < 0.001; ns, not significant; V2, LentiCRISPR V2 vector; sgGPX4, GPX4 knockout.

The aforementioned results showed that the inhibitory effect of Se@Tri-PTs on ferroptosis is associated with the maintenance of the protein levels of GPX4. We next investigated the effect of GPX4 knockout on Se@Tri-PTs-mediated inhibition of ferroptosis in HK-2 cells. As shown in Fig. 6A, the expression of GPX4 in cells was reduced by ~95% after its knockout by sgRNA. CCK-8 assay showed that GPX4 knockout (sgGPX4) could decrease cell viability, enhance the effect of RSL3, and also reverse the inhibition of Se@Tri-PTs in RSL3-treated cells (Fig. 6B). In line with the result of cell viability, Se@Tri-PTs suppressed RSL3-induced cell death and production of lipid ROS, which were markedly reversed by GPX4 knockout (Fig. 6C and D). These results indicated that GPX4 is required for Se@Tri-PTs-mediated inhibition of ferroptosis in HK-2 cells.

### Deficiency of DUSP1 reverses the inhibitory effect of Se@Tri-PTs on ferroptosis

**Fig. 7.**
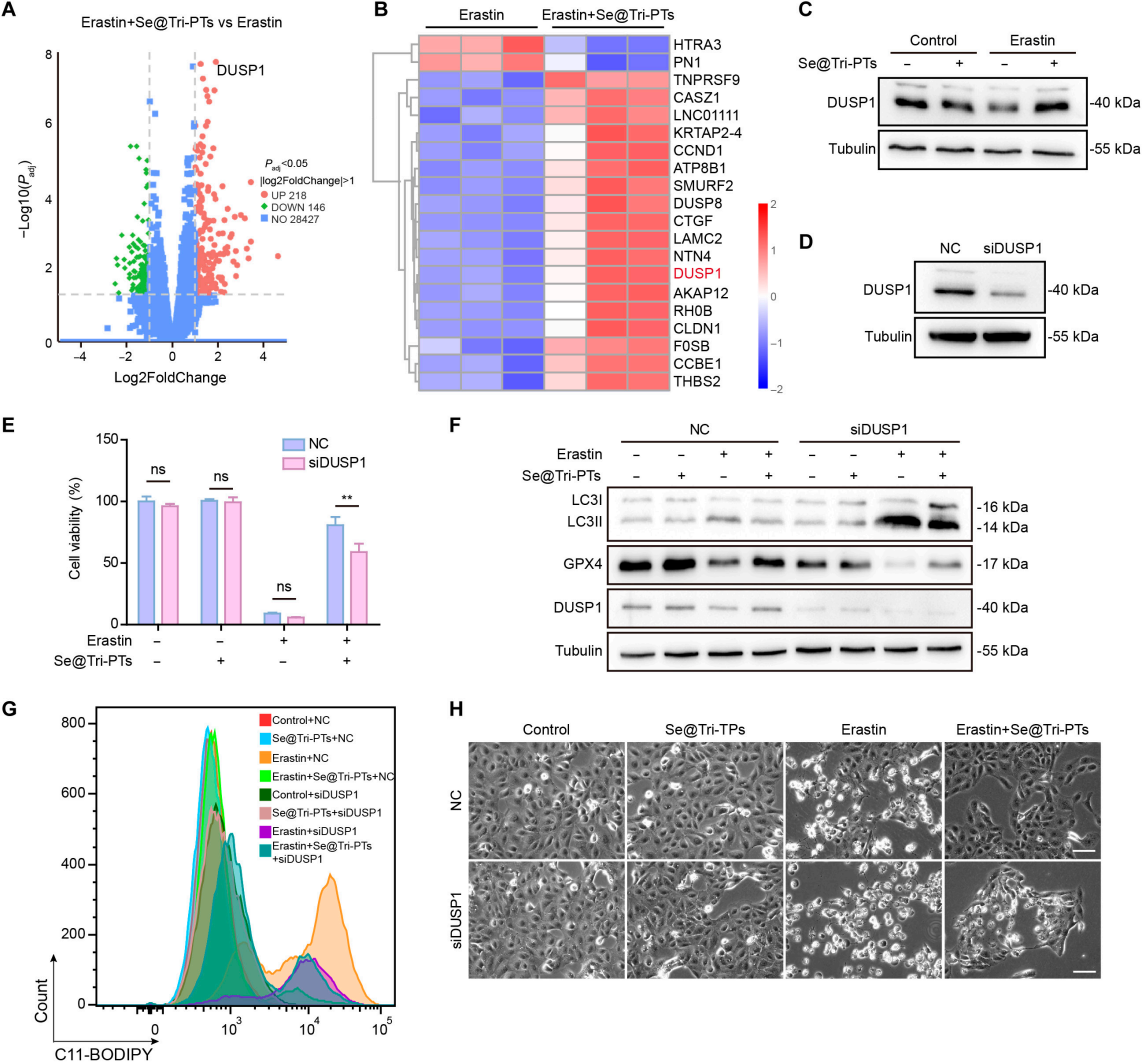
The inhibitory effect of Se@Tri-PTs on ferroptosis depends on the expression of DUSP1. (A and B) Gene expression changes in erastin + Se@Tri-PTs-treated group compared with erastin group are assessed by RNA sequencing and displayed in the volcano plot (A) or heatmap (B). The HK-2 cells were treated with erastin (2 μM) with or without Se@Tri-PTs for 24 h. (C) Western blot analysis was used to detect the expression of DUSP1. (D) Knockdown of DUSP1 by siRNA in HK-2 cells, and the protein level of DUSP1 was detected by Western blotting. (E) Cell viability assayed by CCK-8. (F) Western blotting was used to assess the expression of proteins. (G) Lipid ROS levels were assayed by flow cytometry with the C11-BODIPY probe. (H) The representative live images showing the levels of cell death (scale bar: 50 μm). Data are expressed as mean ± SD (*n* = 3); **P < 0.01; ns, not significant; NC, negative control; siDUSP1, DUSP1 knockdown.

To further explore the molecular mechanism for Se@Tri-PTs to inhibit autophagy in erastin-induced HK-2 cells, we conducted RNA sequencing analysis. The results showed that *DUSP1* was the most markedly up-regulated in Se@Tri-PTs and erastin cotreated group among all the up-regulated genes compared to the erastin group (Fig. 7A and B), and DUSP1 has been reported to regulate ferroptosis via autophagy. Consistent with this, Western blotting results showed that erastin treatment decreased the protein levels of DUSP1, while Se@Tri-PTs recovered DUSP1 expression in erastin-induced HK-2 cells (Fig. 7C). To confirm the effect of DUSP1 on Se@Tri-PTs-mediated inhibition of ferroptosis, DUSP1 was knocked down by siRNA. Figure 7D shows that the expression of DUSP1 protein was reduced approximately 85% after knockdown. Consistent with the results of autophagy activator rapamycin, DUSP1 knockdown significantly reversed Se@Tri-PTs-mediated inhibition of erastin-induced cell viability decrease (Fig. 7E). The Se@Tri-PTs-mediated decrease in LC3II levels and increase in GPX4 levels were reversed by DUSP1 knockdown (Fig. 7F). Furthermore, the suppression of lipid ROS production and cell death by Se@Tri-PTs cotreatment was also markedly reversed by DUSP1 knockdown (Fig. 7G and H). In addition, we also detected the role of AMPK/mTOR signaling in Se@Tri-PTs-mediated inhibition of ferroptosis. The results showed that Se@Tri-PTs had no effects on AMPK/mTOR signaling (Fig. S2). Altogether, these results suggest that DUSP1 plays a key role in Se@Tri-PTs-mediated inhibition of ferroptosis, which is not associated with the AMPK/mTOR pathway.

### Se@Tri-PTs ameliorate FA-induced AKI by inhibiting ferroptosis

**Fig. 8.**
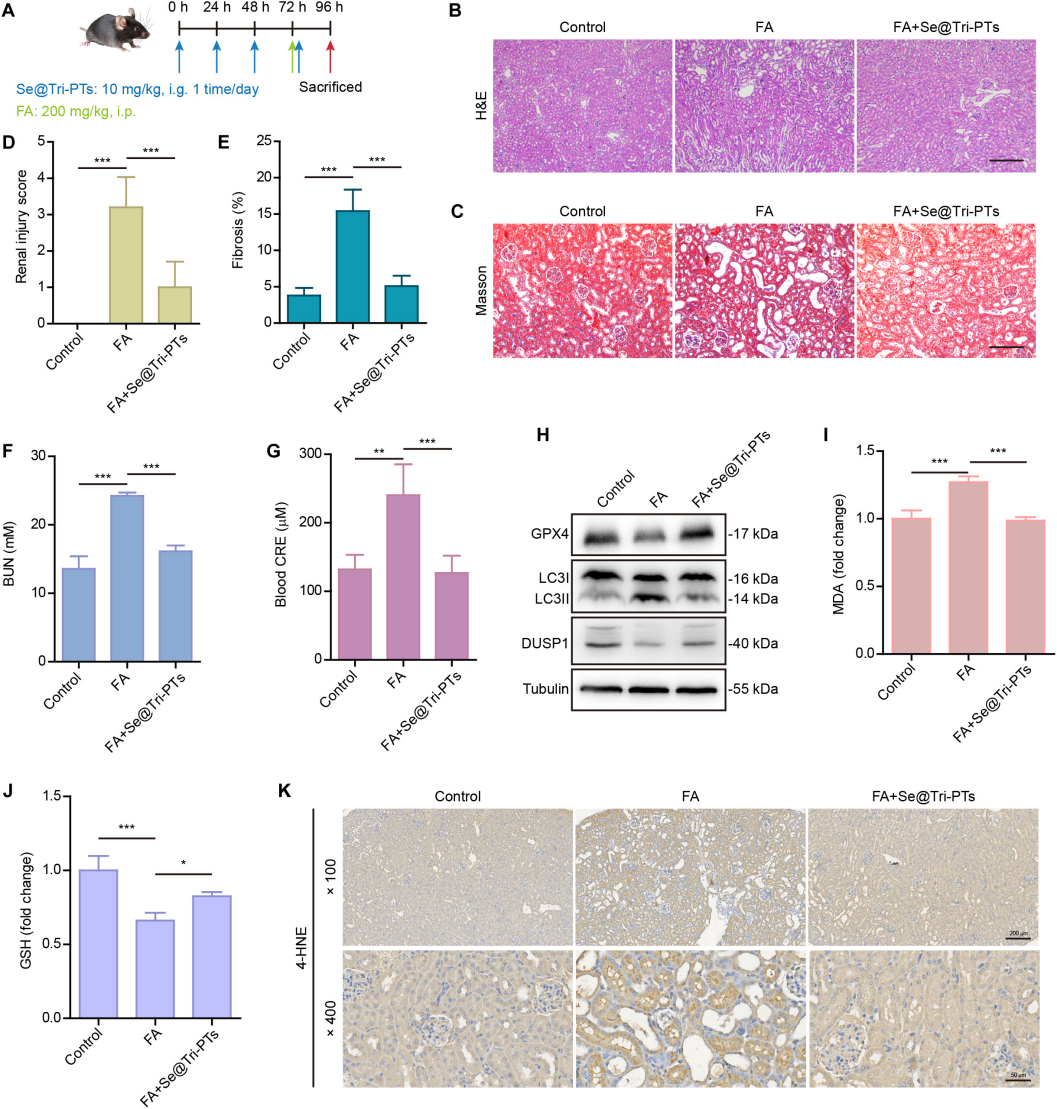
Se@Tri-PTs attenuate kidney injury and ferroptosis in FA-induced AKI mice. (A) The schematic shows the drug dosage and administration schedule of the FA-induced AKI model. (B) The representative histopathological images of kidney after staining by H&E (scale bar: 50 μm). (C) Masson staining was used to analyze the kidney (scale bar: 50 μm). (D) Quantification of renal tubular injury score (*n* = 5) in (B). (E) Quantifications renal fibrosis in (C). (F and G) Analysis of BUN (F) and blood CRE levels (G) is shown by histograms. (H) The expression levels of indicated proteins in the kidney samples were detected by Western blotting. (I) Lipid peroxidation levels in the kidney were detected by MDA assay. (J) GSH levels in the kidney tissues were measured. (K) Representative images of IHC staining for 4-HNE in the kidney tissues (scale bar: 50 μm). Data are expressed as mean ± SD (*n* =5). *P < 0.05; **P < 0.01; ***P < 0.001; FA, folic acid. Se@Tri-PTs, selenized tripterine phytosomes.

As ferroptosis has a critical role in AKI progression, we lastly explored whether Se@Tri-PTs could ameliorate kidney injury and ferroptosis in a mouse model of FA-induced AKI. Mice were intragastrically administered once for 3 consecutive days with Se@Tri-PTs (10 mg/kg body weight) or vehicle (PBS) before a single intraperitoneal injection of FA (200 mg/kg) or NaHCO_3_ solution. One hour after injection of FA, mice were intragastrically administered once again with Se@Tri-PTs or vehicle, and then the mice were sacrificed after 24 h. Consistent with the in vitro results, Fig. 8A shows the experimental procedure of FA-induced AKI and dosage regimen of Se@Tri-PTs. The up-regulation of blood urea nitrogen (BUN) and blood creatinine (CRE) levels are the most routinely parameters of AKI in clinical settings, so we firstly measured the levels of BUN and CRE in the serum of mouse model to assess the affect of Se@Tri-PTs on AKI. Our data showed that the FA injection elevated the levels of BUN and blood CRE in mice (Fig. 8F and G). However, Se@Tri-PTs administration reversed the increase of FA-induced BUN and blood CRE, indicating that Se@Tri-PTs had a protective effect on FA-induced AKI. Furthermore, tubular injury was observed by Masson and H&E staining in the kidney sections. Figure 8B to E show that FA administration resulted in obvious histopathologic injury and fibrosis, including increased interstitial space and severe nephron loss, but Se@Tri-PTs significantly reduced the renal damage. H&E staining also showed that Se@Tri-PTs had no obvious toxicity to other organs including the liver, heart, spleen, and lung (Fig. S3). Western blotting showed that FA decreased the expression levels of GPX4 and DUSP1, and increased the expression levels of LC3II, but Se@Tri-PTs reversed the effects of FA on these 3 proteins (Fig. 8H). We subsequently detected the lipid peroxidation level in the kidney tissue. The concentration of MDA, a final product of lipid peroxidation, was significantly elevated in the FA group, whereas it was efficiently inhibited by Se@Tri-PTs (Fig. 8I). In line with this, Se@Tri-PTs also inhibited FA-caused down-regulation of GSH (Fig. 8J). In addition, immunohistochemistry staining demonstrated that the levels of 4-hydroxy-2-nonenals (4-HNE), another product of lipid peroxidation and a surrogate marker for ferroptosis, were significantly regulated up by FA treatment in the renal tissues. However, Se@Tri-PTs decreased the production of 4-HNE to similar levels of controls (Fig. 8K). Taken together, these data illustrate that Se@Tri-PTs can inhibit FA-induced ferroptosis and mitigate kidney injury in the FA-induced AKI mouse model.

## Discussion

In this study, we developed selenized phytosomes to orally deliver tripterine in an attempt to overcome shortcomings and potentiate its curative effects on high-risk kidney diseases. Prominently, the novel lipid-based formulation enhanced the antiferroptotic activity of tripterine in synergy with selenium in ferroptosis cellular models. Se@Tri-PTs were also able to inhibit ferroptosis in vivo, which was proved to be associated with reduced autophagy-mediated degradation of GPX4 by maintaining DUSP1 expression. Thus, our findings provide a new insight into the action mechanism of Se@Tri-PTs in treating nephropathy by regulating ferroptosis in renal tubular cells.

Presently, 2 classes of small-molecule ferroptosis agonists have been reported: one induces ferroptosis by inhibiting xCT on the cell membrane to decrease intracellular GSH and limit the function of GPX4 on converting lipid hydroperoxides into lipid alcohols by GSH, including erastin; the other directly acts on GPX4, such as RSL3 [24]. Therefore, erastin and RSL3 are commonly used as inducers for inducing ferroptosis in cellular models. In this study, our results showed that erastin or RSL3 induced ferroptosis and production of lipid ROS, which were robustly inhibited by Se@Tri-PTs. Se@Tri-PTs were also able to reverse erastin-mediated decrease of GSH levels. Moreover, Se@Tri-PTs-mediated suppression of ferroptosis was shown to be related to the maintenance of GPX4 protein levels, as evidenced by the knockout of GPX4 in HK-2 cells.

GPX4, a main oxidoreductase to scavenge lipid peroxides using GSH as a reducing agent, acts as an important regulator of ferroptosis [25,26]. GPX4 plays a critical role in converting lipid hydroperoxides into nontoxic lipid alcohols. Maintaining the function of GPX4 is therefore central to limiting ferroptosis. For the SCL7A11/GSH/GPX4 axis, any step of the process is disrupted, and the activity or expression of GPX4 will decrease, resulting in the accumulation of lipid peroxides and contributing to ferroptosis [25]. The SCL7A11 inhibitor erastin or GPX4 inhibitor RSL3 or knockout of GPX4 can enhance cell lipid peroxidation and thereby induce ferroptosis. Some studies reported that the transcription factor nuclear factor erythroid-2 related factor 2 (Nrf2) is an essential regulatory factor in GPX4-dependent ferroptosis pathway, which can up-regulate the activity or expression of GPX4 by enhancing the expression of SCL7A11 to inhibit ferroptosis [27]. A new study also revealed that tripterine could inhibit ferroptosis through the Nrf2/GPX4 pathway [14]. However, we found that Se@Tri-PTs could block the decrease of GPX4 protein expression in erastin or RSL3-stimulated cells, rather than influencing the protein expression of SCL7A11. Meanwhile, Se@Tri-PTs also did not alter the level of GPX4 mRNA, manifesting that Se@Tri-PTs exert their antiferroptotic effect by means of maintaining the protein level of GPX4. Although our findings are inconsistent with the work of others, it might suggest the existence of other regulatory mechanisms for Se@Tri-PTs. Some studies have reported that blocking degradation of GPX4 contributes to the enhancement of cell resistance to ferroptosis, and autophagosomes are accumulated during the process of ferroptosis, implying that autophagy may be involved in the development of ferroptosis [5]. For example, increasing the levels of LAMP2A and CMA leads to the degradation of GPX4 that contributes to ferroptosis, while the degradation of GPX4 protein is retarded by HSPA5 (heat shock protein family A [Hsp70] member 5) [28]. Other reports also showed that classical ferroptosis activators (like erastin and RSL3) were able to increase autophagic flux in AKI [29,30]. Erastin or RSL3 treatment resulted in ferroptosis accompanied by an increase in levels of LC3II and autophagy [31]. Consistent with these findings, our study showed that erastin or RSL3 treatment up-regulated the levels of LC3II and autophagy, whereas Se@Tri-PTs inhibited the up-regulation of these induced by erastin or RSL3, while maintaining the levels of GPX4 protein. However, the autophagy agonist rapamycin reversed Se@Tri-PTs-mediated inhibition of ferroptosis and GPX4 degradation in erastin or RSL3-stimulated cells, suggesting that Se@Tri-PTs could block the degradation of GPX4 by inhibiting autophagy, thereby inhibiting ferroptosis. Of note, copper stress can also induce autophagic degradation of GPX4 and derive ferroptosis [6], indicating that autophagy can directly degrade GPX4 and does not always depend on a selective autophagy (like CMA), which corroborates our results as well.

Selenium (Se), a trace element integral to human and mammalian physiology, exhibits a triad of biological functions: nutritional, toxicological, and detoxifying properties. It is often termed a “life protector” due to roles such as mitigating cisplatin-induced toxicity in organs including the kidneys and the gastrointestinal tract. As an essential trace element enriched in renal tissue, Se incorporates into selenoproteins—notably glutathione peroxidase—where it critically supports antioxidant defense systems and maintains redox homeostasis, thereby protecting renal integrity [32]. For example, Se treatment increased transcription of several selenoprotein genes, including GPX4 [33]. However, our data showed that Se@Tri-PTs did not affect the mRNA expression of GPX4. Some studies also showed that Se supplementation plays a protective role by loading selenocysteine cotranslationally [34]. In our study, Se@Tri-PTs treatment alone could not change the protein level of GPX4. Although we did not use Se treatment as a control, these data indicated that Se@Tri-PTs-mediated increase of GPX4 via protein degradation inhibition (like suppressing autophagy) in erastin or RSL3 treatment cells.

The underlying mechanism of Se@Tri-PTs-mediated autophagy inhibition is unclear in terms of ferroptosis. The regulatory mechanisms of autophagy are complex, and its upstream signaling pathway mainly involves 2 different forms: one is the mTOR-dependent pathway; the other is the mTOR-independent pathway, including AMPK, phosphoinositide 3-kinase (PI3K), mitogen-activated protein kinase (MAPK), extracellular signal-regulated kinase (ERK), phosphatase and tensin homolog (PTEN), and endoplasmic reticulum stress [35]. These regulators control autophagy through their phosphorylation levels [36]. DUSP1 (a threonine-tyrosine dual-specificity phosphatase) takes part in the dephosphorylation and inactivation of MAPKs, ERK, and JNK. One previous study found that DUSP1 knockdown by shRNA increased both basal and rapamycin-stimulated autophagic flux, whereas overexpression of DUSP1 produced the opposite effect, indicating that DUSP1 deficiency induces autophagy [37]. Another study revealed that DUSP1 was down-regulated by acute cardiac ischemia/reperfusion injury and loss of DUSP1 led to the activation of mitophagy, during which the reintroduction of DUSP1 alleviated mitophagy [38]. In the present study, we found that Se@Tri-PTs could restore the level of DUSP1 in erastin-induced cells and decrease the expression of LC3II. Knockdown of DUSP1 by siRNA enhanced erastin-induced autophagy and ferroptosis and reversed the inhibitory effect of Se@Tri-PTs on erastin-induced autophagy and ferroptosis. Additionally, the decrease of DUSP1 levels was induced by FA in mice, while Se@Tri-PTs treatment prevented FA-induced down-regulation of DUSP1. Furthermore, we also found that Se@Tri-PTs had no effects on AMPK/mTOR signaling. In further support of our work, knockdown of DUSP1 increased erastin- or RSL3-induced autophagy-dependent ferroptosis in vitro and in vivo [39]. Although the action mechanism of DUSP1-mediated autophagy remains largely unknown, DUSP1 may offer a new possibility for the therapy of ferroptosis-mediated disease by limiting autophagy.

Recently, with escalating attention focusing on the fundamental roles of ferroptosis in AKI, researchers believe that ferroptosis is involved in the occurrence and development of AKI [40]. For example, defect of ACSL4 (a key enzyme in promoting ferroptosis) significantly inhibited ferroptosis and alleviated the pathological injury of AKI [41]. Conversely, the ACSL4 inhibitor rosiglitazone remarkably ameliorated AKI [41]. Due to the low levels of early intraoperative iron-binding proteins, the capacity of rapidly handling catalytic iron released during extracorporeal circulation was damaged, which caused kidney injury [42]. In ischemia–reperfusion injury (IRI), iron-dependent ferroptosis directly caused synchronized necrosis of renal tubules, and ferroptosis inhibitors exhibited the strong protective effect on IRI [43]. Additionally, one previous study revealed that ferroptosis, but not necroptosis, was important in nephrotoxic FA-induced AKI, in which high levels of lipid peroxidation were monitored in FA-induced AKI and Fer-1 administration alleviated the stress and injury of the kidney [44]. Although the potential pathogenic mechanisms have not been clarified, several key links have been disclosed between ferroptosis and AKI. Consistent with this, our findings show that Se@Tri-PTs can decrease the levels of MDA and 4-HNE by inhibiting autophagy-mediated degradation of GPX4 in FA-induced AKI.

The discovery of different molecular pathways regulating ferroptosis has contributed to the improvement of the understanding of this form of cell death. Evidence for implication of ferroptosis in various diseases (such as AKI, hepatic injury, and neurodegenerative diseases) is increasingly convincing [45]. Therefore, the development of novel ferroptosis inhibitors has been a hot area of research. Meanwhile, multiple novel inhibitors on the basis of the initially discovered inhibitors Fer-1 and Lip-1 are used to address different ferroptosis-driven diseases in vivo, but there are always some limitations, such as low activity [46]. Of note, the active components of traditional medicine have gradually attracted the attention of researchers, and some have shown significant protective effects on ferroptosis-mediated AKI by antioxidation and others. Thus, these active components exhibit huge potential for blocking the progression of AKI. For instance, quercetin alleviates AKI by inhibiting the chemotaxis of macrophages induced by ferroptosis [47]. Nuciferine can alleviate FA-induced AKI by reducing the accumulation of lipid ROS and iron in vitro and in vivo [22]. Tripterine is considered as a therapeutic candidate for AKI. However, the practical application of tripterine is largely limited by its biopharmaceutical properties [48]. To this end, great efforts have been made by practitioners to address the issue. Various nanocarrier-based delivery systems have been explored for tripterine [49], such as micelles, lipid nanoparticles, microemulsions, and liposomes, which significantly improve the delivery or therapeutic efficacy of tripterine. Nevertheless, the development of preferred delivery systems remains an obligation to potentiate the curative outcomes. In our previous studies, selenium-functionalized phytosomes were exclusively developed for the oral or cytoplasmic delivery of tripterine [17,18]. Selenized phytosomes exhibited stronger structural stability and more stable drug loading than conventional lipid nanocarriers [17,18]. The superiority of drug delivery efficiency has been confirmed. In the present study, we further demonstrate that Se@Tri-PTs display more potent pharmacological activity and broader application scenarios. As uncovered in this study, Se@Tri-PTs can effectively alleviate AKI by inhibiting ferroptosis likely owing to the synergy between selenium and tripterine.

In conclusion, this work aimed to engineer Tri nanomedicine (Se@Tri-PTs) and verify its inhibitory effect on AKI involved in ferroptosis in vitro and in vivo. Our data show that the inhibitory effect of Se@Tri-PTs on ferroptosis is prominent and that the underlying mechanism is mediated by inhibiting the degradation of GPX4 via the suppression of autophagy through maintaining DUSP1 expression. Interestingly, Se@Tri-PTs also alleviate FA-induced ferroptosis and AKI in vivo. These findings highlight the potential application of Se@Tri-PTs for the treatment of AKI. Meanwhile, the present study also provides a notion of developing composite drugs that integrate phytomedicine and selenium into applicative nanomedicine to synergistically mediate ferroptosis-associated diseases.

## Data Availability

The data that support the findings of this study are available from the corresponding authors upon reasonable request.
