# Supplementary material for: Selenized Tripterine Phytosomes Alleviate Ferroptosis-Mediated Acute Kidney Injury by Suppressing GPX4 Degradation via the DUSP1/Autophagy Pathway
Source: Biomater Res. 2025 Aug 12;29:0236. doi: 10.34133/bmr.0236 (PMC12343027; doi:10.34133/bmr.0236)
Supplement: Supplementary 1 — Figs. S1 to S3 [file bmr.0236.f1.docx]

**Selenized Tripterine Phytosomes Alleviate Ferroptosis-Mediated Acute Kidney Injury by Suppressing GPX4 Degradation Via the DUSP1/Autophagy pathway**

Liang Yan^1,2,3,4,†^, Qi Feng^5,†^, Yong Sun^4^, Bo-ning Zeng^6^, Chuan-chuan Sun^7^, Qing-bing Zha^1, 3,*^, Xing-wang Zhang^8,*^, Shi-ping Zhu^2,*^





**Figure S1 Se@Tri-PTs had no effect on SLC7A11 levels.** (A-B) Western blotting was used to detect the expression of SLC7A11.





**Figure S2 Se@Tri-PTs inhibited-autophagy is uncorrelated with AMPK/mTOR signaling. (**A-B**)** Analysis of the expression of indicated by western blotting.


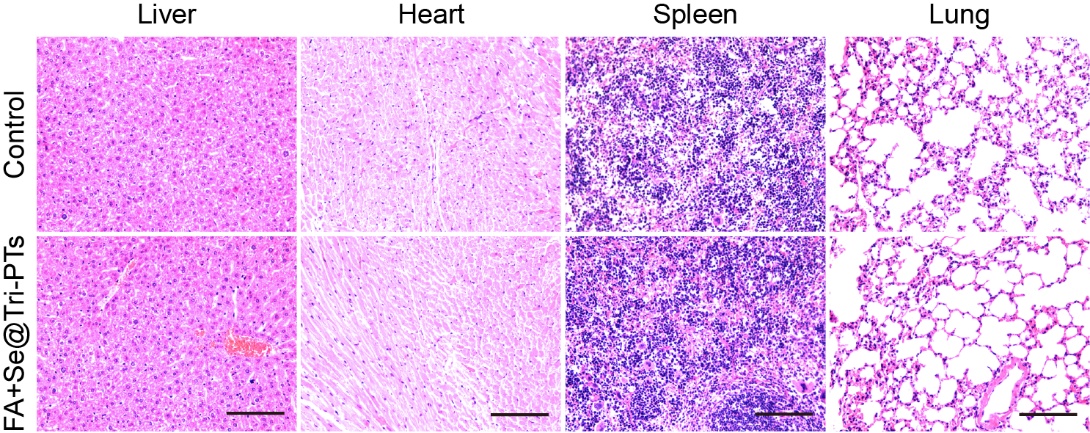


**Figure S3 Se@Tri-PTs had no obvious toxicity to other organs.** H&E staining was used to analyze the effects of Se@Tri-PTs in multiple organs (scale bar 50 μm).
